# Occluded Coronary Artery among Non-ST Elevation Myocardial Infarction Patients in Department of Cardiology of a Tertiary Care Centre: A Descriptive Cross-sectional Study

**DOI:** 10.31729/jnma.7934

**Published:** 2023-01-31

**Authors:** Manju Sharma, Raja Ram Khanal, Sangam Shah, Ratna Mani Gajurel, Chandra Mani Poudel, Suman Adhikari, Vijay Yadav, Surya Devkota, Shovit Thapa

**Affiliations:** 1 Department of Cardiology, Manmohan Cardio Thoracic Vascular and Transplant Centre, Institute of Medicine, Maharajgunj, Kathmandu, Nepal; 2 Maharajgunj Medical Campus, Institute of Medicine, Tribhuvan University, Maharajgunj, Kathmandu, Nepal; 3 Department of Cardiology, Chitwan Medical College, Bharatpur, Nepal

**Keywords:** *coronary angiography*, *MINOCA*, *Non-ST elevation myocardial infarction*

## Abstract

**Introduction:**

Non-ST elevation myocardial infarction is frequently thought to be caused by incomplete blockage of the culprit artery, whereas ST elevation myocardial infarction is frequently thought to be caused by total occlusion of the culprit artery. The objective of the study was to find out the prevalence of occluded coronary arteries among non-ST elevation myocardial infarction patients department of cardiology of a tertiary care centre.

**Methods:**

A descriptive cross-sectional study was conducted among non-ST elevation myocardial infarction patients in a tertiary care centre from 22 June 2020 to 21 June 2021 after taking ethical approval from the Institutional Review Committee [Reference number: 4271 (6-11) E2 076/077]. A total of 196 patients were included in the study by simple randomized sampling. Data on the patient's clinical profile, angiographic findings, and in-hospital complications were recorded. Point estimate and 95% Confidence Interval were calculated.

**Results:**

Among 126 non-ST elevation myocardial infarction patients included in the study, the prevalence of occluded coronary artery was 41 (32.54%) (24.36-40.72, 95% Confidence Interval).

**Conclusions:**

The prevalence of occluded coronary arteries was similar to the studies done in similar settings.

## INTRODUCTION

Non-ST Elevation Myocardial Infarction (NSTEMI) is frequently thought to be caused by incomplete blockage of the culprit artery, whereas STEMI is frequently thought to be caused by total occlusion of the culprit artery.^1-6^ According to research, around a quarter of NSTEMI, is caused by full occlusion of the culprit artery, which is identical to the findings of STEMI on coronary angiography.^7,8^ Despite this, NSTEMI occluded artery is frequently viewed as a less serious condition than STEMI. There was very little data on the differences in clinical features and outcomes between NSTEMI with the occluded artery (NSTEMIOA) and NSTEMI with patent artery/non-occluded (NSTEMIPA), especially in the context of early vs late percutaneous revascularization.

The objective of the study was to find out the prevalence of occluded coronary arteries among non-ST elevation myocardial infarction patients department of cardiology of a tertiary care centre.

## METHODS

This was a descriptive cross-sectional study conducted at a tertiary hospital of Manmohan cardiothoracic vascular and transplant centre (MCVTC) with a primary percutaneous coronary intervention (PCI) facility.

The patients with ages greater than 18 years old and NSTEMI who underwent coronary angiography from 22 June 2020 to 21 June 2021 were included in the study. The Institutional Review Committee [Reference number: 4271 (6-11) E2 076/077)] of the institute of medicine approved the study. The sample size was calculated using


$$\begin{array}{ll} \text{n} & ={\text{Z}}^{2}\times \frac{\text{p}\times \text{q}}{{\text{e}}^{\text{2}}}\\ & =1.96^{2}\times \frac{0.90\times 0.10}{0.07^{2}}\\ & =71 \end{array}$$

However, after clearing the data only 126 met the inclusion criteria.

Where,

n = minimum required sample size
Z = 1.96 at 95% of Confidence Interval (CI)
p = prevalence of occluded coronary arteries in NSTEMI patients, 90%^9^
q = 1-p
e = margin of error, 7%

The patients with ST-elevation myocardial infarction, left bundle branch block (LBBB), and troponin I rise following PCI/coronary artery bypass graft surgery (CABG) were included from the study. Similarly, patients with NSTEMI who did not undergo coronary angiography and patients who did not give written consent were also excluded. An occluded coronary artery was defined as the presence of a lesion with 100% stenosis or thrombolysis in myocardial infarction (TIMI) flow grade 0 to 1 in one or more major coronary vessels on invasive coronary angiography. Major branch occlusion was incorporated into the major vessel territory. A non-occluded or patent coronary artery was defined as TIMI flow grade 2/3. The culprit artery of the NSTEMI was determined by the cardiologist performing the coronary angiography based on the findings of electrocardiogram (ECG) changes, angiography, and echocardiography. After ascertaining the severity of coronary artery disease, the mode of revascularization is determined and if PCI is indicated, the procedure is done, and the patient is shifted to the coronary care unit (CCU) for observation and further management. Sometimes, thrombosuction and plain old balloon angioplasty (POBA) may also be done according to requirement. In the case of complex lesions, the revascularization procedure (PCI/CABG) is decided by the heart team approach.

Data were collected from patients using questionnaires, physical examination, investigation parameters (cardiac biomarkers, ECG, echocardiography), coronary angiographic, and revascularization details, and in-hospital complications. Data were compiled, edited, and checked to maintain consistency. The data were recorded in a Microsoft Excel 2014 and analyzed using IBM SPSS Statistics version 24.0. Point estimate and 95% CI were calculated.

## RESULTS

A total of 126 patients were included with the diagnosis of NSTEMI who underwent coronary angiography during the study period. Among 126 NSTEMI patients, the prevalence of occluded coronary arteries (OCA) was 41 (32.54%) (24.36-40.72, 95% CI). However, in patients with OCA, there was female predominance. The mean age of presentation in patients with occluded coronary arteries was 61.02±14.16 years.

Dyslipidemia was present in 15 (36.59%) NSTEMI patients with occluded coronary arteries. The baseline characteristics of the study population and NSTEMI patients with occluded arteries presented about 24 hours after the development of symptoms (Table 1).

**Table 1:** Baseline characteristics of the study population (n= 41).

Characteristics	n (%)
Age (years), mean ± SD	61.02±14.16
Sex	
Male	16 (39.02)
Female	25 (60.98)
Smoking	
Smoker	19 (46.34)
Non smoker	22 (53.66)
Hypertension	32 (78.05)
Diabetes	20 (48.78)
Dyslipidemia	15 (36.59)
Family history of CAD	11 (26.83)
Body Mass Index (BMI)	25.91±3.27
Abdominal circumference	83.00±7.33
Prior History of MI	12 (29.27)
Chronic Kidney Disease (CKD)	5 (12.20)
MHD	1 (2.44)
Previous PCI	3 (7.32)
Previous CABG	-
Duration of symptoms in hours (Median)	24 (58.54)
Angina	37 (90.24)
Dyspnea	27 (65.85)
Syncope	1(2.44)
Palpitation	2 (4.88)
Nausea & vomiting	5(12.20)
Epigastric pain	1 (2.44)

The mean level of Troponin I was 6.59±12.58. Heart failure developed in 17 (41.46%) of NSTEMI patients with occluded arteries. The in-hospital mortality was 1(2.44%) patient with OCA and the baseline laboratory results and complications developed among patients (Table 2).

**Table 2:** Biochemical characteristics and in-hospital complications in patients with NSTEMI (n= 41).

Characteristics	n (%)
Total Cholesterol	161.25±5.02
HDL	36.56±8.54
LDL	98.21±33.89
Triglyceridemia	170.68±104.45
Creatinine	1.00±0.44
Troponin I	3.56±3.64
**ECG findings**	
ST-depression	3 (7.32)
T-wave inversion	16 (39.02)
ST-depression and T wave inversion	5 (12.20)
LBBB	3 (7.32)
RBBB	4 (9.76)
Paroxysmal Supraventricular	-
Tachycardia (PSVT)	
No ischemic changes	13 (31.71)
Echo-LV function	
Normal (>50%)	13 (31.71)
LV dysfunction (<50%)	28 (68.29)
Heart failure	17 (41.46)
Cardiogenic Shock	1 (2.44)
Arrythmia	1 (2.44)
Atrial fibrilation	1(2.44)
Ventricular Tachycardia/Ventricular	-
fibrilation (VT/VF)	
PSVT	-
MR	3 (7.32)
Pericardial effusion/pericarditis	3 (7.32)
AKI	4 (9.76)
In-hospital mortality	1 (2.44)

TVD was also the predominant lesion in NSTEMI with the occluded artery 18 (43.90%). In overall NSTEMI patients culprit artery was as follows: circumflex branch of left coronary artery (LCX) 20 (48.78%), RCA 6 (14.63%) (Table 3).

**Table 3:** Coronary angiography and interventions in patients with NSTEMI (n= 41).

Characteristics	n (%)
Dominance	
Right	35 (85.37)
Left	5 (12.20)
Co-dominant	1 (2.44)
CAG result	
Normal	-
Minor CAD	-
SVD	10 (24.39)
DVD	11(26.83)
TVD	18 (43.90)
LM	2 (4.88)
Culprit Vessel	
LAD	13 (31.71)
LCx	20 (48.78)
RCA	6 (14.63)
RI	2 (4.88)
Treatment strategy	
Conservative	14 (34.15)
Routine PCI	25 (60.98)
CABG	4 (9.76)
Multi vessel PCI	14 (34.15)

## DISCUSSION

In this study out of 126 patients, 32.50% were NSTEMI with occluded coronary arteries while 67.5% were NSTEMI with non-occluded coronary arteries.

In our study, 32.50% of NSTEMI patients had occluded coronary arteries which were similar to previously conducted studies.^10^ In one study done in the USA, the frequency of NSTEMI with occluded coronary artery was around 24% but the study population was limited only to patients undergoing PCI. Further studies have shown variable findings and, depending upon the difference in patient selection, the percentage of occluded coronaries was found to be around 29% to 63%.^11^ The reason for having higher OCA prevalence in our study might be a late presentation of patients to a medical facility, prior undetected MI, or missed STEMI due to a lack of early identification of ischemic heart disease.

There was male predominance (59.5%) in our study presenting with NSTEMI. Also, the majority of the patients were male in NSTEMI with the occluded coronary artery (61% vs 39%). Men have a 2.4-fold overall risk for NSTEMI compared with women.^12-13^ The proportion of female patients being lower compared to male patients in our study may be due to fewer females attending hospitals for medical aid, tolerance of symptoms, and less likely to have coronary angiography due to gender-based inequalities in treatment intensity of NSTEMI.^14^

Studies have shown that the south Asian population had a higher rate of MI at a younger age (mean age 53 years) compared to the western population explained by multiple risk factors at ages<60 years.^15,16^ However, our study showed that the mean age of presentation was 65 years. Large-scale studies are required to explain this difference. On the contrary, patients with occluded coronary arteries were younger in our study similar to the other study.^17^

Hypertension, diabetes mellitus, smoking and dyslipidemia are the major risk factors for NSTEMI. In our study, a family history of CAD was found to be statistically significant. Family history has been emphasized as a major nonmodifiable risk factor by the National Cholesterol Education Program Adult Treatment Panel (NCEP-ATP) III guidelines.^18^ Family history of MI is an important risk marker for increased MI risk, and particular weight should be placed on the number of affected first- and second-degree relatives and the age of the relative at the time of presentation. According to the Danish national health registers, a detailed family history could be very useful in assessing MI risk, especially in persons aged 35-55 years.^19^

In our study, NSTEMI patients with occluded coronary arteries presented earlier to the hospital in comparison to non-occluded coronary artery patients. However, there was still a delay in the presentation (>24 hours). This delay in a presentation can lead to delayed revascularization in an occluded artery which can lead to both early and long-term adverse outcomes. The Occluded Artery Trial (OAT), showed no benefit to revascularization for patients with an occluded infarct artery 24 hours after symptom onset.^20^ The enhanced survival in STEMI patients by rapid reperfusion of the infarct-related artery was noted compared to NSTEMI patients.^21^ Therefore, these NSTEMI patients with an occluded coronary artery may represent STEMI equivalents who could benefit from earlier revascularization.

There were some limitations of our study. Our study population was small and hence it was difficult to achieve statistical significance for the differences in clinical outcomes between NSTEMI patients with OCA and NOCA. Our study was also single-centred and hence the results obtained may not be generalized to other populations. Follow up study was not done in our study as a result long-term effect of revascularization could not be ascertained so a study with long-term follow-up is required.

## CONCLUSIONS

The prevalence of occluded coronary arteries was similar to the studies done in similar settings. There is as yet no reliable tool to identify this group of patients before performing angiography. These patients have similar presentations and angiographic findings as ST-segment Elevation myocardial infarction. So whether timely reperfusion will benefit this group of patients requires further studies.
